# Secular trends in chronic respiratory diseases mortality in Brazil, Russia, China, and South Africa: a comparative study across main BRICS countries from 1990 to 2019

**DOI:** 10.1186/s12889-021-12484-z

**Published:** 2022-01-13

**Authors:** Jianjun Bai, Yudi Zhao, Donghui Yang, Yudiyang Ma, Chuanhua Yu

**Affiliations:** 1 Department of Epidemiology and Biostatistics, School of Public Health, Wuhan University, 185# Donghu Road, 430072 Wuhan, China; 2 Global Health Institute, Wuhan University, 185# Donghu Road, 430072 Wuhan, China

**Keywords:** Chronic respiratory diseases (CRD), Chronic obstructive pulmonary disease (COPD), Asthma, Mortality, Age-period-cohort model, BRICS

## Abstract

**Background:**

As the emerging economies, the BRICS (Brazil, Russia, India, China, and South Africa) shared 61.58% of the global chronic respiratory diseases (CRD) deaths in 2017. This study aimed to assess the secular trends in CRD mortality and explore the effects of age, period, and cohort across main BRICS countries.

**Methods:**

Data were obtained from the Global Burden of Disease Study (GBD) 2019 and analyzed using the age-period-cohort (APC) model to estimate period and cohort effects between 1990 and 2019. The net drifts, local drifts, longitudinal age curves, period/cohort rate ratios (RRs) were obtained through the APC model.

**Results:**

In 2019, the CRD deaths across the BRICS were 2.39 (95%UI 1.95 to 2.84) million, accounting for 60.07% of global CRD deaths. Chronic obstructive pulmonary disease (COPD) and asthma remained the leading causes of CRD deaths. The age-standardized mortality rates (ASMR) have declined across the BRICS since 1990, with the most apparent decline in China. Meanwhile, the downward trends in CRD death counts were observed in China and Russia. The overall net drifts per year were obvious in China (-5.89%; -6.06% to -5.71%), and the local drift values were all below zero in all age groups for both sexes. The age effect of CRD presented increase with age, and the period and cohort RRs were following downward trends over time across countries. Similar trends were observed in COPD and asthma. The improvement of CRD mortality was the most obvious in China, especially in period and cohort effects. While South Africa showed the most rapid increase with age across all CRD categories, and the period and cohort effects were flat.

**Conclusions:**

BRICS accounted for a large proportion of CRD deaths, with China and India alone contributing more than half of the global CRD deaths. However, the declines in ASMR and improvements of period and cohort effects have been observed in both sexes and all age groups across main BRICS countries. China stands out for its remarkable reduction in CRD mortality and its experience may help reduce the burden of CRD in developing countries.

**Supplementary Information:**

The online version contains supplementary material available at 10.1186/s12889-021-12484-z.

## Background

Over the recent decades, epidemiologic evidences have shown that non-communicable diseases (NCD) are being the main contributors to disease burden worldwide [1, 2]. As a crucial category of NCDs, chronic respiratory diseases (CRD) have been the third leading cause of deaths globally, ranked behind cardiovascular diseases and cancers [3]. According to Global Burden of Diseases, Injuries, and Risk Factors (GBD) 2019, CRD caused 3.91 million deaths in 2017 [4], which led to a heavy burden on individuals and health systems, especially for low- and middle-income countries.

As the economies coalition composed of developing countries, Brazil, Russia, India, China, and South Africa (BRICS) has undergone the rapid economic expansion, and the consequent population swell, environmental pollution, health resources inequalities, and transition in disease burden to NCDs [5, 6]. Although socioeconomic disparities and CRD prevention of the BRICS countries have improved during the recent years [7, 8], the burden of CRD remains heavy across the BRICS, with 2.41 million deaths from CRD in 2017, accounting for 61.58% of CRD deaths worldwide [9–11]. Given that other developing countries may encounter the similar dilemma along the epidemiological and economic transition, advances in the BRICS health policies and CRD prevention and control is instructive for low- and middle-income countries [12].

To improve people’s respiratory health, governments and non-governmental organizations have made great efforts over the past decades. In 2013, BRICS committed to strengthen intra-BRICS cooperation for improving breath health. In 2015, the United Nations adopted Sustainable Development Goals (SDG) target 3.4 to reduce premature mortality from NCDs by a third by 2030, including CRD [13]. In 2017, BRICS reaffirmed to make efforts to achieve the SDGs 2030[14]. Hence, to better understand the gap between current conditions and SDGs, it is essential to estimate the time trends of the CRD burden over recent period among the BRICS by a comparable manner. To our knowledge, there is no study available giving combined comparison of the disease burden in CRD across the BRICS.

In this study, we adopted the data from GBD 2019 to examine the secular trends of CRD mortality and assessed the effects of age, period, and birth cohort across the BRICS using age–period–cohort (APC) model. Previous analyses have focused on CRD mortality trends over time by age and region, but ignored the accumulation of health risks since birth. Crude mortality calculated by traditional statistical methods is incapable of eliminating or controlling the interaction between age, period, and birth cohort factors. It cannot accurately reflect the actual effect of age, period, and birth cohort on mortality [15]. While assessing the relative contribution of period and cohort effects to overall temporal trends might help determine the efficacy of early policy initiatives and identify future priorities.

The findings of this study may provide new evidence of influencing factors for CRD and help public health managers to assess the impact of previous interventions and formulate further policies.

## Methods

### Data sources

We obtained the population, deaths, and age-standardized mortality rates (ASMR) data for all age and age-standardized groups across the BRICS from GBD 2019, which provideds comparable and systematic standardized estimations of CRD for 204 countries and territories, by age and sex, from 1990 to 2019. Original data sources for Brazil were primary from the Mortality Information System [10]; for Russia, from center for Demographic Research, New Economic School (Russia) [16]; for China, from censuses, the Disease Surveillance Point system, Maternal and Child Surveillance System, and Chinese Center for Disease Control and Prevention Cause of Death Reporting System [17]; for South Africa, largely from South Africa Vital Registration system and Statistics South Africa [18]. All the relevant estimations are publicly available online and all downloaded from Global Health Data Exchange (GHDx) query tool (http://ghdx.healthdata.org/gbd-results-tool). The Cause of Death Ensemble model (CODEm), a highly systematised tool, was used to estimate mortality based on original data sources and covariates, including socio-demographic Index, lagging distribution income, education, alcohol consumption and so on. DisMod-MR2.1, based on Bayesian meta-regression tool, evaluate the available data and ensure the consistency between epidemiological parameters [2, 19]. CRD and its subtypes (COPD, pneumoconiosis, asthma, interstitial lung disease, and pulmonary sarcoidosis) were defined based on Ninth and Tenth Revision of the International Classification of Diseases [2, 3, 19]. Rates were age-standardized by the GBD 2019 global standard population.

### Statistical analysis

The age–period–cohort (APC) model can evaluate the effect of age, period, and birth cohort on the mortality of chronic diseases and has been widely used to analyze the mortality trends of NCDs [20, 21]. We adopt the APC Web Tool for parameter estimation along with associated statistical hypothesis tests. The tool can be accessed at http://analy-sistools.nci.nih.gov/apc/, and its codes were open at https://github.com/CBIIT/nci-webtools-dceg-age-period-cohort.

The CRD age-standardized mortality rates were appropriately categorized into consecutive 5-year periods from 1990 to 2019, successive 5-year age groups from 20 to 24 years to 80-84 years, and the corresponding consecutive 18 birth cohorts (1910-1914, 1915-1919, …, 1995-1999). Besides, we take central age groups (50-54 years), 2000-2004 period, and 1950-1954 cohort as reference [22, 23].

The following parameters were obtained through the APC model. The net drifts represent the log-linear trends by period and birth cohort, showing the overall estimated annual percentage change in the ASMR. The local drifts represent the log-linear trends by period and birth cohort for specific age groups, indicating estimated annual percentage change over time specific to the age group. The longitudinal age curves represent fitted longitudinal age-specific rates in reference cohort adjusted for period deviations. The period rate ratios (RR) are the ratio of age-specific rates in a specific period relative to the reference period. The cohort rate ratios (RRs) are the ratio of age-specific rates in each cohort relative to reference cohort [24]. The period effects represent variations in different screening methods, medical technology applications, or disease classification standards over specific periods. Cohort effects refer to the long-term trend of disease mortality of those born around the same time, which may be related to lifestyle, external environment, and risk factors exposure.

Since the three parameters of the APC model have exact linear dependency (period=age +cohort), it’s difficult to calculate the only model estimator. Therefore, we adopted the APC web tool with the intrinsic estimator method to address the multicollinearity problem[23]. Wald chi-square tests were carried out to verify the significance of estimable parameters and functions (Additional file 1: Supplementary Table 1). We used general linear models to evaluate the interaction terms between sex and calendar year/birth cohort and assess the statistical significance of the slopes for the period/cohort RRs. Statistical analyses were performed using SPSS software. Statistical significance was considered when a two-sided p ≤ 0.05.

## Results

### Deaths from CRD

**Fig. 1:**
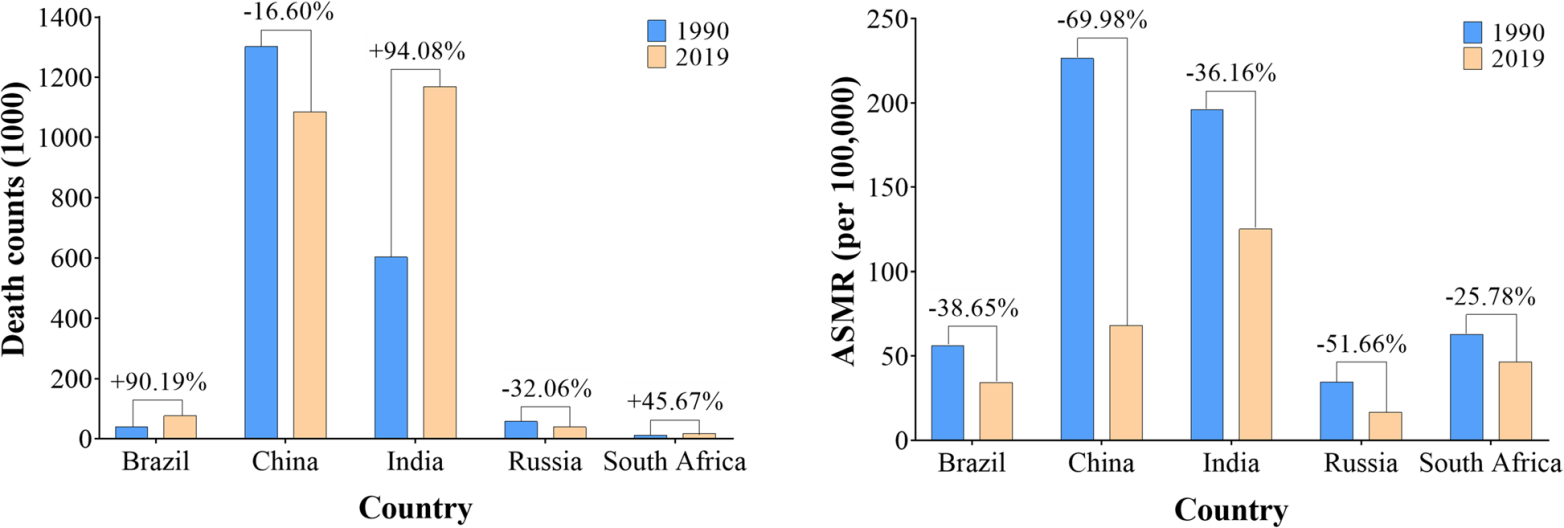
The CRD deaths, age-standardized mortality rates (ASMR) and percentage changes across BRICS countries

According to the GBD 2019, the total deaths caused by CRD in BRICS were 2.39 (95%UI 1.95 to 2.84) million in 2019, accounting 60.07% of the CRD deaths globally (3.97 [3.58 to 4.30] million). Across the BRICS, the deaths of CRD varied considerably, with the most deaths observed in India (1.17 [0.90 to 1.36] million), followed by China (1.09 [0.93 to 1.32] million). These two countries recorded 56.71% of global deaths attributable to CRD in 2019. However from 1990 to 2019, an obvious improvement in deaths was seen in Chinese patients with CRD, whereas patients in India showed worsening health (Fig. 1). Besides, encouraging is that the ASMRs had fallen across the BRICS, with the most apparent decline in China, from 226.43 (95%UI 170.16 to 251.43) to 67.98 (57.80 to 83.43) per 100,000, followed by Russia.

COPD and asthma remained the two leading causes of CRD deaths during study period (Additional file 3: Supplementary Fig. 1). In 2019, 2.05 (1.68 to 2.46) million people died from COPD and 232.85 (158.12 to 313.84) thousand deaths caused by asthma across the BRICS, accounting for 86.00% and 9.75% of the total number of deaths from CRD in BRICS.

### Net drifts and local drifts for CRD mortality across the BRICS

The net drifts were similar in Brazil (-2.24%, 95% CI -2.31% to -2.17%) and South Africa (-2.33%, -2.68% to -1.97%). The net drifts in China and Russia were -5.89% (-6.06% to -5.71%) and -3.82% (-4.08% to -3.56%), representing the relatively more significant reductions in CRD mortality. Also, the CRD mortality for both sexes had achieved relatively great improvement across countries (Table 1).

The local drifts were all below zero by age group among four countries, indicating the improvements in CRD mortality. And the most remarkable progress was detected in China for all age groups. The most impressive improvements were for Chinese aged 55 to 59 (-6.45%), Russian aged 55 to 59 (-4.67%), Brazilian aged 50 to 54 (-2.23%), and South African aged 20 to 24 (-5.13%). The local drifts by sex were similar to the overall trends (Additional file 2: Supplementary Table 2).

**Table 1:** The net drifts and local drifts for Brazil, China, Russia, and South Africa (%)

Sex	Net drifts^a^(95%CI)			
	Brazil	China	Russia	South Africa
Both	-2.24 (-2.31 , -2.17)	-5.89 (-6.06 , -5.71)	-3.82 (-4.08 , -3.56)	-2.33 (-2.68 , -1.97)
Male	-2.32 (-2.41 , -2.23)	-5.24 (-5.4 , -5.08)	-3.99 (-4.31 , -3.67)	-2.13 (-2.41 , -1.84)
Female	-2.07 (-2.13 , -2.02)	-6.89 (-7.2 , -6.57)	-3.57 (-3.8 , -3.34)	-2.49 (-3.00 , -1.98)
Age	Local drifts^a^(95%CI)			
	Brazil	China	Russia	South Africa
20-24	-1.90(-2.34 , -1.46)^a^	-5.93(-7.54 , -4.29)	-4.09(-6.54 , -1.57)	-5.13(-7.35 , -2.85)
25-29	-2.15(-2.46 , -1.84)^a^	-6.20(-7.14 , -5.25)	-3.33(-4.70 , -1.94)	-4.86(-6.20 , -3.49)
30-34	-2.33(-2.57 , -2.10)^a^	-6.00(-6.68 , -5.32)	-2.91(-3.84 , -1.97)	-4.04(-5.06 , -3.00)
35-39	-2.38(-2.57 , -2.18)	-5.92(-6.43 , -5.40)	-3.10(-3.81 , -2.39)	-3.31(-4.19 , -2.43)
40-44	-2.33(-2.48 , -2.17)	-5.94(-6.30 , -5.58)	-3.75(-4.32 , -3.19)	-2.84(-3.60 , -2.08)
45-49	-2.25(-2.37 , -2.12)	-6.13(-6.38 , -5.87)	-4.28(-4.71 , -3.84)	-2.61(-3.28 , -1.94)
50-54	-2.23(-2.33 , -2.13)	-6.37(-6.57 , -6.18)	-4.56(-4.87 , -4.25)	-2.18(-2.75 , -1.60)
55-59	-2.29(-2.37 , -2.21)	-6.45(-6.61 , -6.30)	-4.67(-4.89 , -4.45)	-1.50(-2.00 , -1.00)
60-64	-2.36(-2.43 , -2.29)	-6.12(-6.23 , -6.01)	-4.50(-4.67 , -4.32)	-1.11(-1.56 , -0.66)
65-69	-2.32(-2.38 , -2.26)	-5.71(-5.8 0, -5.62)	-3.89(-4.05 , -3.74)	-0.88(-1.28 , -0.47)
70-74	-2.16(-2.21 , -2.11)	-5.23(-5.30 , -5.16)	-3.10(-3.26 , -2.94)	-1.04(-1.43 , -0.66)
75-79	-2.00(-2.06 , -1.95)	-4.72(-4.79 , -4.66)	-2.57(-2.73 , -2.41)	-1.10(-1.51 , -0.68)
80-84	-1.97(-2.04 , -1.90)	-4.23(-4.31 , -4.14)	-2.69(-2.89 , -2.49)	-1.28(-1.84 , -0.73)

Note: ^a^ *CI* confidence interval; *Net drifts* represent the overall annual percentage change in the age-standardized rate based on period and birth cohort. *Local drifts* indicate the annual percentage change over time specific to the age group. All of *Net drifts* and *Local drifts* were statistically significant (p < 0.05). India was excluded because India’s data didn’t meet the APC model’s requirements

### The age, period, and cohort effects on CRD mortality

**Fig. 2:**
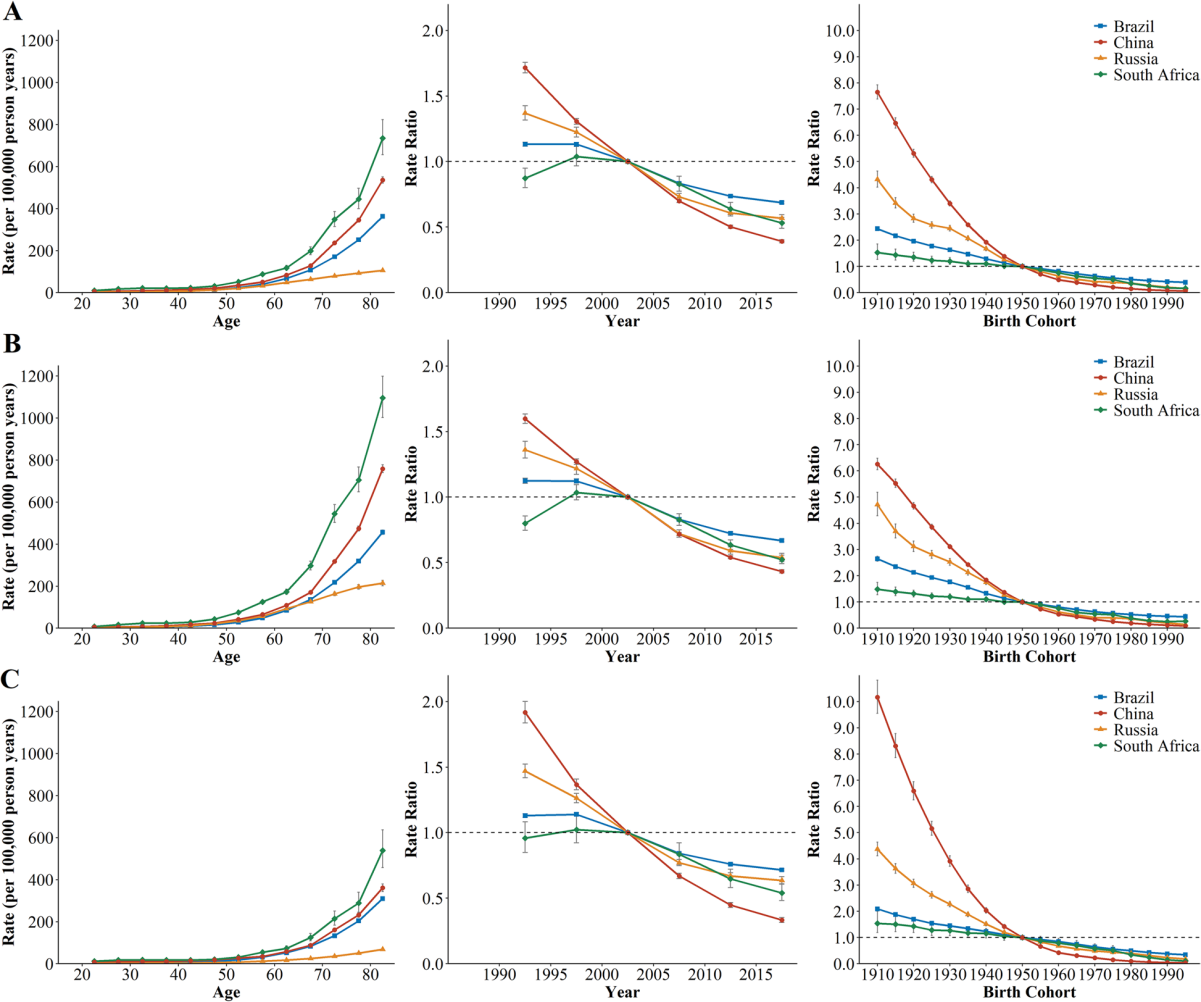
The age, period, and cohort effects on CRD mortality across BRICS countries; **A** for both sexes; **B** for male; **C** for female

Figure 2 illustrates the longitudinal age curves of sex-specific CRD mortality. After controlling for period and cohort effects, we found that the distribution of CRD mortality with age exhibited exponential distribution. The CRD mortality showed the rapidly expanding trends from 45 to 49 age group to 80-84 age group. Among the four countries, South Africa showed the steepest increases with age, from 10.27 to 734.64 per 100,000, followed by China. In comparison, the increase of CRD mortality in Russia was relatively moderate, from 2.40 to 105.72 per 100,000. In each age group, the CRD mortality of males were higher than those of females in China, Russia, and South Africa (P < 0.05). The sex disparity of mortality gradually increased with age.

The estimated period RRs showed prominent downward trends for both sexes across four countries (Fig. 2). The most obvious improvement of CRD risk was detected in China, with period RRs decreasing from 1.72 to 0.39, followed by Russia, from 1.37 to 0.57. Besides, the improvements in CRD risk were more significant for Chinese females. Similarly, the estimated cohort RRs followed the monotonic decreasing trends for both sexes across countries, especially for Chinese females, decreased from 10.17 in 1910 to 0.04 in 1995. For China and Russia, the improvements in CRD risk were noticeable for people born before 1960. The advances in Brazil and South Africa were relatively minor but favorable. For South Africa, the period and cohort effects were flat. The cohort RRs were statistically significant for both sexes in four countries based on the results of the general linear model (P<0.001 for all).

### The age, period, and cohort effects on CRD subtype mortality

**Fig. 3:**
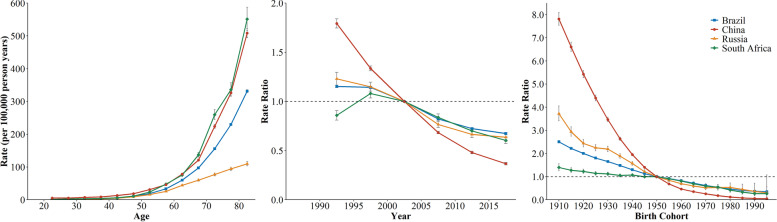
The age, period, and cohort effects on COPD mortality across BRICS countries

COPD was the major contributor to CRD mortality across four countries, and the distribution of COPD mortality with age exhibited the characteristic exponential distribution (Fig. 3). The COPD mortality in South Africa showed the fastest increase with age, reaching the peak of 551.05 per 100,000 at the 80-84 age group. Similar trend was observed in China, climbing from 5.11 to 508.16 per 100,000. Russia showed a relatively moderate increase in COPD mortality. As for period RRs, the most obvious improvement was observed in China, decreasing from 1.79 to 0.37. Russia and Brazil performed similarly and favorably on improvement of period effects. Likewise, the decline of cohort RRs was the most dramatic in China, from 7.82 in 1910 to 0.05 in 1995, followed by Russia, from 3.72 to 0.30.

**Fig. 4:**
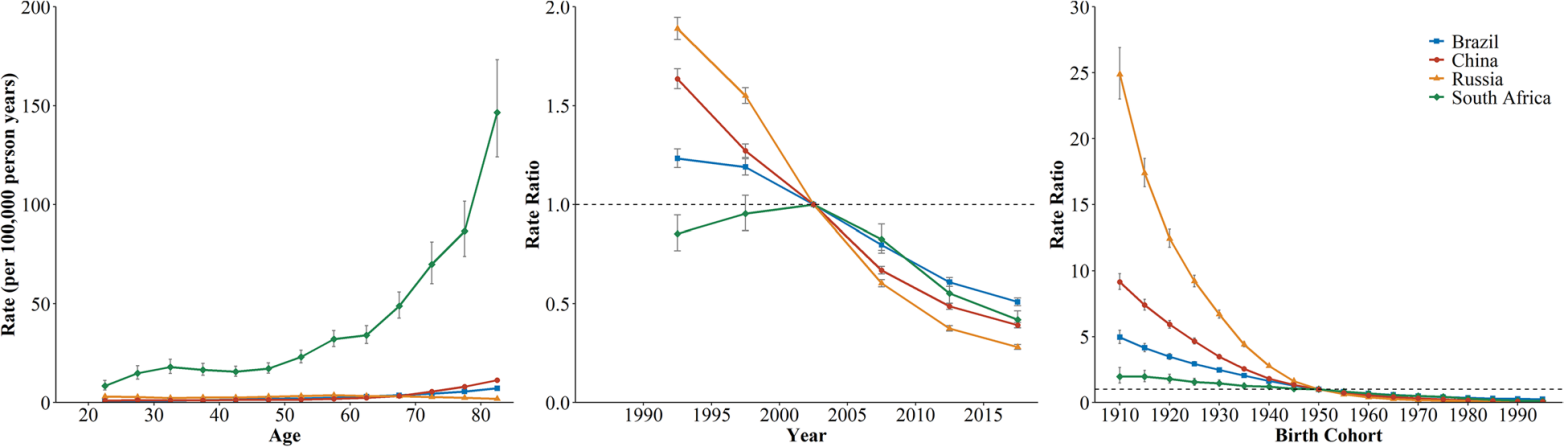
The age, period, and cohort effects on asthma mortality across BRICS countries

As for the second contributor to CRD mortality, the fastest increase of asthma mortality was detected in South Africa, from 8.43 to 146.60 per100,000 (Fig. 4). In China and Brazil, the increase in asthma mortality with age was not obvious. While the asthma mortality in Russia showed the decreasing trend after age 55, falling to 1.89 per100,000 at the 80-84 age group. During the study period, the period and cohort RRs of asthma mortality decreased across four countries. And the declining trends of period and cohort RRs in Russia was most dramatic, followed by China. While the declining trends of asthma in South Africa was flat.

For interstitial lung disease and pulmonary sarcoidosis, the age effect showed a continuous upward trend. South Africa had the highest mortality across all age groups, as well as Brazil. Among four countries, the period and cohort effects were flat, suggesting no improvements for the whole population over study period (Additional file 4: Supplementary Fig. 2). For pneumoconiosis, Russia was excluded since the data didn’t meet the requirements. The age effect showed a continuous upward trend with age. South Africa had surpassed China as the country with the highest mortality after the 50-54 age group. For period and cohort RRs, China had a more pronounced downward trend, which indicated the improvement of pneumoconiosis risk in China (Additional file 5: Supplementary Fig. 3).

## Discussion

According to GBD 2019, CRD caused 3.97 (95% CI 3.58 to 4.30) million deaths worldwide, ranking behind cardiovascular diseases and cancers. The BRICS account for a large proportion of global CRD deaths, and China and India alone have contributed over half of global CRD deaths in 2019. However, encouraging is that the improvements in ASMRs have been seen since 1990 across the BRICS. Meanwhile, declines in CRD deaths have been detected in China and Russia over the study period.

Within the BRICS, there were striking differences between countries both in rates and secular trends. India had the most CRD deaths and the highest CRD mortality in recent years. Meanwhile, with the population growing and aging, the number of CRD deaths in India continued to rise during the study period. Although the improvements in ASMRs have been observed, but CRD burden per person in India was still higher than the global average level [25]. Evidences suggested that the higher CRD burden in India was partly due to late diagnosis and poor management [26–28]. Insufficient clinical knowledge of COPD and asthma, mainly symptom-based diagnosis, inadequate use of gold standard, and overlapping clinical features lead to underdiagnosis or misdiagnosis of CRD in India [26, 27]. Meanwhile, the acceptance of inhaled drugs is relatively low in India [29, 30]. Regional disparities and socioeconomic status disparities also pose challenges to the prevention and treatment of CRD in India.

The greater period and cohort improvements have been observed in China across all categories of CRD mortality, especially for COPD and asthma. China’s investment in health is likely to be a major factor. Over the study period, the enhanced diagnosis, treatment techniques, better access to basic public health services, the new rural cooperative medical system, and the reform of CRD healthcare costs have contributed to improvement of CRD mortality in China[31, 32]. Besides, for main risk factors of CRD, air pollution and tobacco, Chinese government has adopted a series of control measures. Ambient air pollution in most areas of China has gradually improved[33], and exposure reduction of ambient and indoor air pollution were also observed among rural areas[34]. Meanwhile chinese smoking prevalence has declined since 1990 in both sexes [35]. Although favorable decling trends in deaths have been observed, due to population growth and aging, the number of CRD deaths was still higher across the BRCIS. For note, huge population size, rapid aging population, relatively large smoking population, and regional and sex disparities should receive more attention with supportive policies to lessen the premature CRD mortality in China.

Russia had a lower baseline CRD mortality level, and the improvements of period and cohort effects were impressive. The number of CRD deaths and ASMR in Russia both declined from 1990 to 2019. The gains were the result of period and cohort effects, and Russia’s reform in health care may be a main factor. Since 1993, mandatory health insurance reform and pharmaceutical policy reform have focused on improving access and equality to health care, which contributed to the early detection and treatment of CRD [36–38]. Besides, for the major risk factor, Russia has adopted stricter prevention and control measures against smoking. Some studies have predicted that the smoking prevalence in Russia would decrease by as much as 50% by 2055 if the current policies remain unchanged [39, 40]. Meanwhile, given the high correlation between CRD and CVD, intervention in CRD would help reduce the overall disease burden in Russia [41]. However, aging, COPD misdiagnosis, a high proportion of undiagnosed patients, low adherence to treatment, and exposure to occupational risk factors impede further advance in breath health for Russia [16, 42].

From 1990 to 2019, CRD deaths in Brazil rose mainly due to population growth and ageing process, while the trend in CRD mortality declined with improvement over period and birth cohorts. In 1994, Brazil launched the Family Health Program (FHP) and promoted it throughout the country; both the FHP and the subsequent strategies have contributed to prevention, diagnosis, and treatment of NCDs [43]. In 2006, Brazil joined the GARD and provided an excellent platform for CRD preventing and controlling [44]. For smoking, Brazil issued Framework Convention on Tobacco Control in 2006, the Smoke-Free Law in 2014 and subsequent tobacco control policies to reduce tobacco consumption. The tobacco use prevalence decrease by roughly 60% from 1998 to 2013 [45]. As for COPD and asthma, Brazil strengthened the training of general practitioners working at the community level and announced to provide non-paying treatment for severe asthmatic patients and health care facilities, education, and free medication for all asthmatics[44]. For note, with rapid industrialization and urbanization in Brazil, air pollution and occupational exposure are becoming more serious. This would increase the risk of CRD and impact those with pre-existing conditions [10].

The ASMR of CRD in South Africa showed a downward trend; however, compared with other BRICS countries, the period and cohorts effect in South Africa were flat. Over the past years, South Africa has concentrated resources on the treatment of infectious diseases such as AIDS and tuberculosis, and these investments seem to have been rewarded, the increase in life expectancy was observed, but the burden of NCDs has not been contained [46, 47]. Besides, another concern related to CRD in South Africa was aging; with the rapidly growing population aged 60 years and older, aging would lead to dramatic changes in patterns of disease burden and increase the risk of respiratory diseases in South Africa [48, 49]. Furthermore, HIV patients have a higher risk of respiratory diseases among children and adults [50]. Since 1994, South African has committed to promoting tobacco control, but the effect is scant [51]; smoking prevalence has been rising between 2008 and 2011 [52]. Additionally, occupational risk factors have also been major problems in sub-Saharan Africa [53]. Mine-related facilities are the primary source of airborne particulate matter and metals pollution, which would seriously affect the respiratory health of workers and surrounding residents [54, 55]. So from 1973, many efforts have been made up, like the Surveillance of Work-related and Occupational Respiratory Diseases, the South Africa Occupational Diseases in Mines and Works Act, and the Mine Health and Safety Act[56, 57]. Meanwhile, education and training for primary care nurses in South Africa would help them better cope with CRD and has been implemented in primary care clinics across South Africa [58].

There are several limitations to our study. Frist, because the period and age intervals should be fixed and equal in the APC tool, data of people aged ≥85 years could not be analyzed because of the data availability (they were designated as one age group in GBD database). But substantially higher mortality of CRD happens in seniors aged ≥85 years. While in our study, age group–specific annual percent changes over time maintained less than zero, which was just more moderate than that of younger groups. And we could confirm to a certain extent that the declining trends in CRD mortality would remain among seniors aged ≥85 years across BRICS. Meanwhile, previous studies confirmed that the CRD mortality of those aged ≥85 years is decreasing in a similar pattern [4, 10, 59]. Second, GBD studies provide comparable and systematic standardized estimates of global CRD burden, while data on cause of death record, medical death certificate, primary cause of death identification are not available for GBD 2019, which are also references for the comparison of CRD mortality between countries [60–62]. Third, it is evitable that the completeness and accuracy on primary CRD data may cause bias, although many adjusted methods have been conducted by GBD to reduce such bias. Finally, the APC model is based on the population level, so ecological fallacy might occur, the interpretation of the study might not apply to the individual level.

## Conclusions

The burden of chronic respiratory disease remains heavy among the BRICS, with China and India alone contributing more than half of the global CRD deaths. While the ASMRs, period effects, and cohort effects have been declining both in sexes and all age groups from 1990 to 2019. Similar downward trends were observed for the two main subtypes, COPD and asthma. Meanwhile, the age effects suggest that more attention should be paid to the middle-aged and elderly. Among BRICS, China stands out for its remarkable reduction in CRD mortality over time, both in recent birth cohorts and across all age groups. Its example may offer experience for other developing countries in reducing the burden of CRD with rapid economic development.

## Supplementary Information

**Additional file 1: Supplementary table 1.** Wald Chi Square tests for estimable functions in the APC model.

**Additional file 2: Supplementary table 2.** The local drifts (%) for Brazil, China, Russia, and South Africa by sex.

**Additional file 3: Supplementary figure 1.** Proportion of deaths by CRD subtypes between 1990 and 2019.

**Additional file 4: Supplementary figure 2.** The age, period, and cohort effects on interstitial lung disease mortality across main BRICS countries.

**Additional file 5: Supplementary figure 3.** The age, period, and cohort effects on pneumoconiosis mortality across main BRICS countries.

## Data Availability

The datasets generated and/or analysed during the current study are available in the Global Health Data Exchange (GHDx) repository, [http://ghdx.healthdata.org/gbd-results-tool]

## Abbreviations

CRD: Chronic respiratory diseases
COPD: Chronic obstructive pulmonary disease
BRICS: Brazil, Russia, India, China, and South Africa
NCD: Non-communicable diseases
APC: Age-period-cohort
ASMR: Age-standardized mortality rates
RR: Rate ratios
ICD: International Classification of Diseases
SDG: Sustainable Development Goals
GBD: Global Burden of Diseases, Injuries, and Risk Factors
GHDx: Global Health Data Exchange
GARD: Global Alliance against Chronic Respiratory Diseases
