# Supplementary material for: Secular trends in chronic respiratory diseases mortality in Brazil, Russia, China, and South Africa: a comparative study across main BRICS countries from 1990 to 2019
Source: BMC Public Health. 2022 Jan 13;22:91. doi: 10.1186/s12889-021-12484-z (PMC8759233; doi:10.1186/s12889-021-12484-z)
Supplement: Supplementary file 1 — Additional file 1: Supplementary table 1. Wald Chi Square tests for estimable functions in the APC model. [file 12889_2021_12484_MOESM1_ESM.docx]

Table S1. Wald Chi Square tests for estimable functions in the APC model.

| Null Hypothesis | Brazil | | China | | India | | Russia | | South Africa | |
| --- | --- | --- | --- | --- | --- | --- | --- | --- | --- | --- |
|  | Chi-Square | P-Value | Chi-Square | P-Value | Chi-Square | P-Value | Chi-Square | P-Value | Chi-Square | P-Value |
| Net Drift = 0 | 3941.31 | <0.001 | 4244.23 | <0.001 | 336.70 | <0.001 | 798.69 | <0.001 | 158.98 | <0.001 |
| All Period RR = 1 | 4801.50 | <0.001 | 4386.89 | <0.001 | 343.82 | <0.001 | 932.70 | <0.001 | 326.66 | <0.001 |
| All Cohort RR = 1 | 13354.83 | <0.001 | 39170.43 | <0.001 | 1306.56 | <0.001 | 4598.18 | <0.001 | 186.62 | <0.001 |
| All Local Drifts = Net Drift | 108.37 | <0.001 | 843.69 | <0.001 | 18.86 | 0.13 | 311.49 | <0.001 | 53.90 | <0.001 |
