# Supplementary material for: Secular trends in chronic respiratory diseases mortality in Brazil, Russia, China, and South Africa: a comparative study across main BRICS countries from 1990 to 2019
Source: BMC Public Health. 2022 Jan 13;22:91. doi: 10.1186/s12889-021-12484-z (PMC8759233; doi:10.1186/s12889-021-12484-z)
Supplement: Supplementary file 2 — Additional file 2: Supplementary table 2. The local drifts (%) for Brazil, China, Russia, and South Africa by sex. [file 12889_2021_12484_MOESM2_ESM.docx]

Table S2. The local drifts for Brazil, China, Russia, and South Africa by gender (%)

| Age | Brazil-M | Brazil-F | China-M | China-F | Russia-M | Russia-F | South-M | South-F |
| --- | --- | --- | --- | --- | --- | --- | --- | --- |
| 22.5 | -1.41 (-1.99 to -0.83) | -2.51 (-2.88 to -2.14) | -5.09 (-6.57 to -3.58) | -7.13 (-10.07 to -4.09) | -4.24 (-7.42 to -0.95) | -3.94 (-5.82 to -2.02) | -3.36 (-5.18 to -1.50) | -6.85 (-9.94 to -3.66) |
| 27.5 | -1.75 (-2.15 to -1.34) | -2.66 (-2.91 to -2.41) | -5.19 (-6.07 to -4.30) | -7.57 (-9.24 to -5.86) | -3.38 (-5.10 to -1.63) | -3.29 (-4.40 to -2.17) | -3.75 (-4.86 to -2.62) | -5.98 (-7.81 to -4.10) |
| 32.5 | -2.06 (-2.37 to -1.74) | -2.69 (-2.88 to -2.49) | -5.01 (-5.64 to -4.37) | -7.39 (-8.60 to -6.16) | -2.92 (-4.06 to -1.76) | -2.86 (-3.65 to -2.05) | -3.53 (-4.37 to -2.69) | -4.54 (-5.96 to -3.11) |
| 37.5 | -2.23 (-2.49 to -1.98) | -2.56 (-2.71 to -2.40) | -5.09 (-5.57 to -4.61) | -7.15 (-8.08 to -6.22) | -3.14 (-3.99 to -2.28) | -2.91 (-3.55 to -2.27) | -3.12 (-3.83 to -2.41) | -3.43 (-4.66 to -2.18) |
| 42.5 | -2.29 (-2.50 to -2.09) | -2.34 (-2.47 to -2.21) | -5.20 (-5.53 to -4.87) | -7.14 (-7.80 to -6.48) | -3.88 (-4.54 to -3.21) | -3.29 (-3.81 to -2.77) | -2.83 (-3.44 to -2.23) | -2.67 (-3.76 to -1.57) |
| 47.5 | -2.33 (-2.50 to -2.17) | -2.10 (-2.20 to -1.99) | -5.41 (-5.65 to -5.18) | -7.29 (-7.77 to -6.81) | -4.46 (-4.96 to -3.95) | -3.59 (-4.01 to -3.17) | -2.68 (-3.20 to -2.16) | -2.33 (-3.30 to -1.35) |
| 52.5 | -2.41 (-2.54 to -2.28) | -1.92 (-2.01 to -1.84) | -5.73 (-5.90 to -5.55) | -7.41 (-7.78 to -7.04) | -4.78 (-5.13 to -4.42) | -3.81 (-4.12 to -3.49) | -2.25 (-2.69 to -1.80) | -1.97 (-2.82 to -1.11) |
| 57.5 | -2.56 (-2.67 to -2.46) | -1.84 (-1.91 to -1.78) | -5.93 (-6.07 to -5.79) | -7.31 (-7.61 to -7.02) | -4.86 (-5.11 to -4.61) | -3.97 (-4.20 to -3.73) | -1.45 (-1.83 to -1.07) | -1.61 (-2.35 to -0.86) |
| 62.5 | -2.70 (-2.78 to -2.61) | -1.82 (-1.87 to -1.76) | -5.67 (-5.77 to -5.57) | -6.84 (-7.05 to -6.63) | -4.65 (-4.84 to -4.45) | -4.00 (-4.18 to -3.81) | -1.06 (-1.40 to -0.71) | -1.38 (-2.05 to -0.71) |
| 67.5 | -2.68 (-2.75 to -2.61) | -1.73 (-1.78 to -1.68) | -5.28 (-5.36 to -5.20) | -6.40 (-6.57 to -6.24) | -4.16 (-4.34 to -3.98) | -3.90 (-4.05 to -3.74) | -0.89 (-1.20 to -0.57) | -1.05 (-1.65 to -0.44) |
| 72.5 | -2.46 (-2.53 to -2.39) | -1.63 (-1.68 to -1.59) | -4.80 (-4.87 to -4.74) | -5.98 (-6.10 to -5.85) | -3.43 (-3.62 to -3.23) | -3.66 (-3.80 to -3.51) | -1.00 (-1.30 to -0.70) | -1.11 (-1.67 to -0.55) |
| 77.5 | -2.19 (-2.26 to -2.12) | -1.63 (-1.67 to -1.58) | -4.29 (-4.36 to -4.23) | -5.46 (-5.57 to -5.34) | -2.81 (-3.02 to -2.61) | -3.36 (-3.48 to -3.23) | -0.98 (-1.32 to -0.64) | -1.10 (-1.68 to -0.52) |
| 82.5 | -2.04 (-2.14 to -1.94) | -1.75 (-1.81 to -1.68) | -3.73 (-3.82 to -3.65) | -4.93 (-5.07 to -4.80) | -2.94 (-3.22 to -2.67) | -3.24 (-3.39 to -3.10) | -1.14 (-1.61 to -0.67) | -1.13 (-1.86 to -0.40) |
