# Supplementary figures and images for: Secular trends in chronic respiratory diseases mortality in Brazil, Russia, China, and South Africa: a comparative study across main BRICS countries from 1990 to 2019
Source: BMC Public Health. 2022 Jan 13;22:91. doi: 10.1186/s12889-021-12484-z (PMC8759233; doi:10.1186/s12889-021-12484-z)

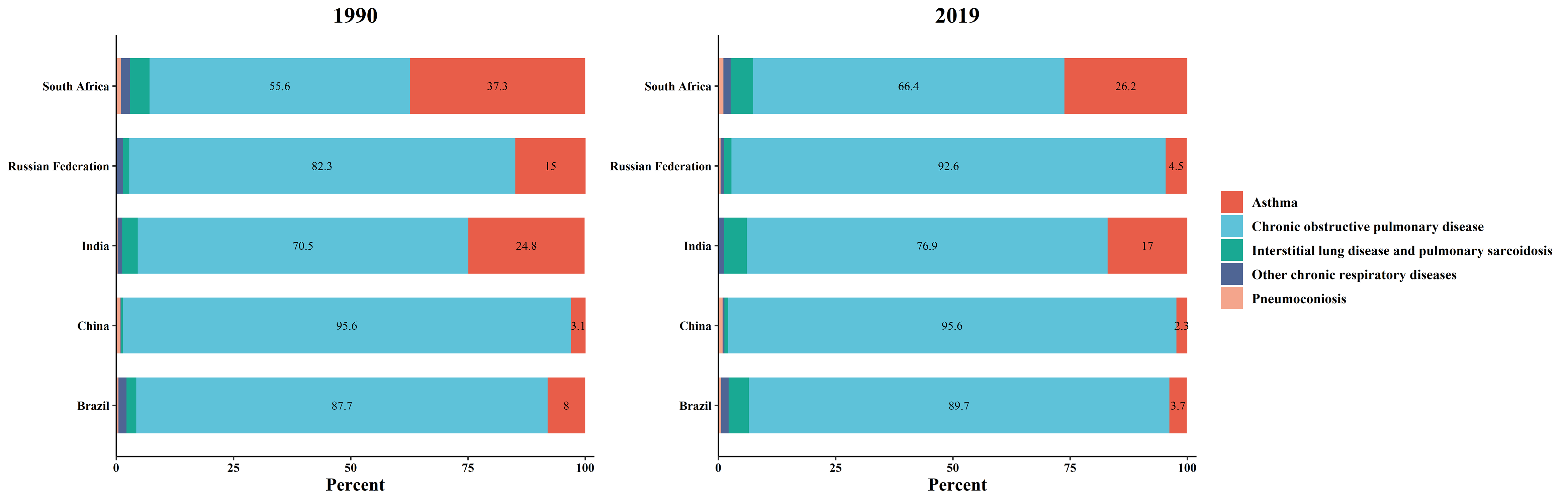

Supplement: Supplementary file 3 — Additional file 3: Supplementary figure 1. Proportion of deaths by CRD subtypes between 1990 and 2019. [file 12889_2021_12484_MOESM3_ESM.tif]

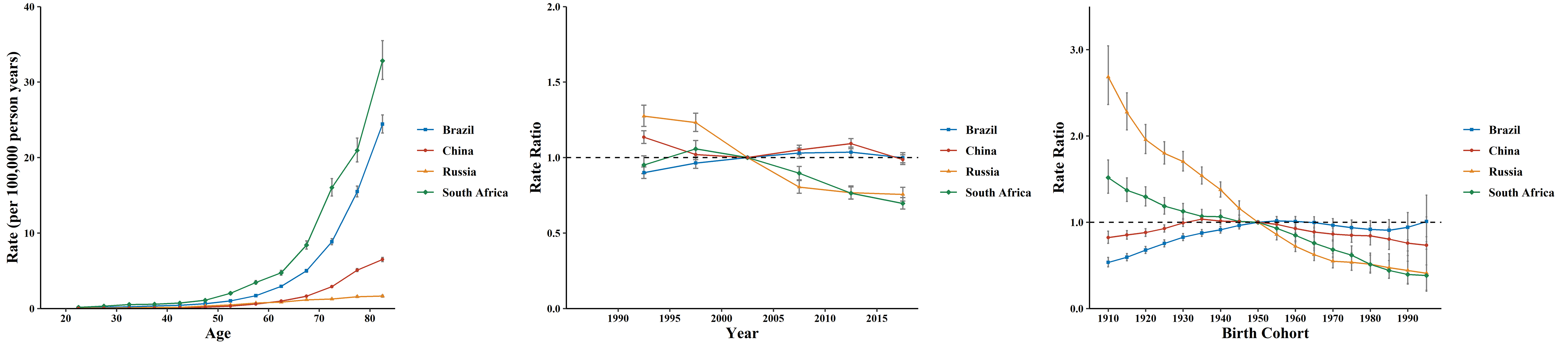

Supplement: Supplementary file 4 — Additional file 4: Supplementary figure 2. The age, period, and cohort effects on interstitial lung disease mortality across main BRICS countries. [file 12889_2021_12484_MOESM4_ESM.tif]

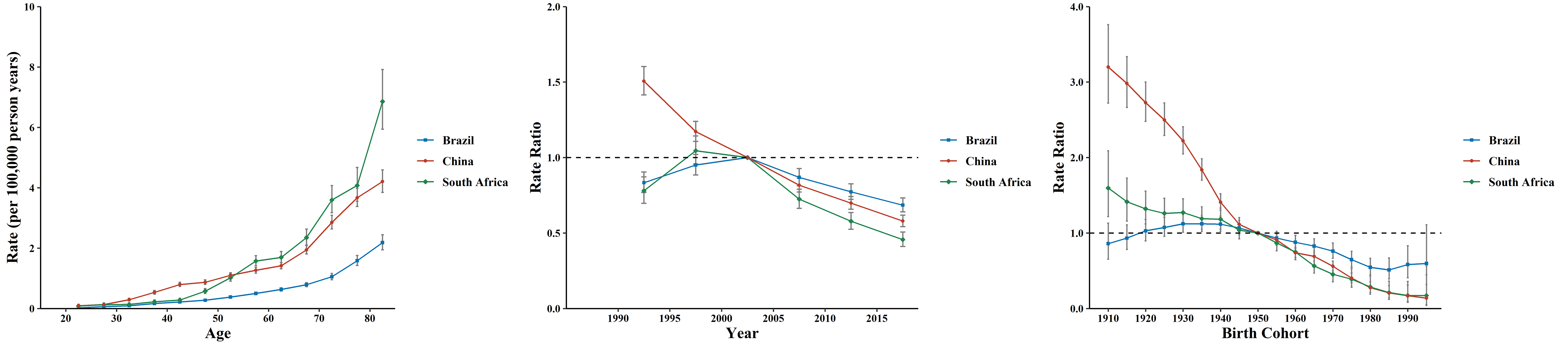

Supplement: Supplementary file 5 — Additional file 5: Supplementary figure 3. The age, period, and cohort effects on pneumoconiosis mortality across main BRICS countries. [file 12889_2021_12484_MOESM5_ESM.tif]
